# Severe propylthiouracil-induced hepatotoxicity in pregnancy managed successfully by liver transplantation: A case report

**DOI:** 10.1186/1752-1947-5-461

**Published:** 2011-09-19

**Authors:** Evan Sequeira, Sikolia Wanyonyi, Raj Dodia

**Affiliations:** 1 Aga Khan University Hospital, PO Box 30270, Nairobi 00100 GPO, Kenya

## Abstract

**Introduction:**

Propylthiouracil-induced severe hepatotoxicity is a relatively rare occurrence, with very few cases reported in the literature. The management of this complication in pregnancy can be a challenge because of the effects of the various treatment options on the fetus.

**Case presentation:**

We report a rare case of fulminant hepatic failure in a 36-year-old gravida 2 black woman of African descent that occurred at 17 weeks gestation following propylthiouracil treatment for Graves' disease. Her liver failure was managed by liver transplantation and thyroidectomy. Her pregnancy was continued to term, though with not so favorable early childhood sequelae.

**Conclusion:**

This case illustrates a very rare complication of treatment with a presumed safe drug during pregnancy followed by adverse neonatal outcomes due to the extensive treatment.

## Introduction

Propylthiouracil (PTU)-induced severe hepatotoxicity is a relatively rare occurrence, despite observed transient increases in liver function test values at the initiation of treatment [1,2]. A one in 10,000 incidence of liver failure following PTU treatment has been reported in the United States of America [3]. Fulminant hepatic failure during pregnancy occurs more frequently in the third trimester of gestation, and its etiology usually derives from obstetric-related conditions and/or infectious diseases [4,5]. Management of this condition poses an ethical dilemma, as the clinician has to choose between maternal well-being and fetal health. We report a rare case of fulminant hepatic failure in the second trimester following PTU treatment for severe thyrotoxicosis in pregnancy that was successfully managed by liver transplantation and thyroidectomy with a good outcome for the mother but unfavorable neonatal sequelae.

## Case presentation

A 36-year-old para 1+0, gravida 2{previous caesarean delivery due to fetal distress, no miscarriages/abortion, with one living child} black woman of African descent was diagnosed with Graves' thyrotoxicosis at seven weeks gestation. Her antenatal profile had been unremarkable and she did not have any comorbidities. There was no relevant family history, and she neither smoked cigarettes nor consumed alcohol. Her previous pregnancy had been uneventful, and she had delivered a healthy baby normally. She presented to our hospital with unexplained palpitations, tachycardia, and excessive sweating. Her examination revealed that she had obvious proptosis. Her cardiovascular and respiratory examinations were normal. We ordered thyroid function tests in view of this presentation. Her thyroid-stimulating hormone level was 0.06 μU/mL (normal range, 0.27 to 4.2 μU/mL), free tri-idothyronine (T3) was 21.08 pg/mL (normal range in first trimester, 2.5 to 3.9 pg/mL), and free thyroxine (T4) was 54.39 ng/mL (normal range in first trimester, 0.9 to 1.5 ng/mL). Her antenatal booking tests and ultrasonography were unremarkable. At 10 weeks gestation, following consultation with an endocrinologist, she was commenced on PTU 100 mg thrice daily, which led to significant improvement in her thyroid function tests. At 17 weeks gestation, she developed scleral icterus. Upon further evaluation at a hospital in the US, a diagnosis of fulminant hepatitis was made. Conservative management with plasmapharesis and intravenous steroids was attempted, but her condition deteriorated to stage IV liver failure, necessitating intensive care with ventilatory support. Subsequently, a liver transplant was performed at 18 weeks gestation, followed by a thyroidectomy two weeks later.

After undergoing transplantation, she was continued on immunosuppressive therapy with tacrolimus, cyclosporine, prednisolone, and thyroid hormone replacement with levothyroxine. Her antenatal care was continued in Nairobi, Kenya, after her discharge from the hospital in the US.

Ultrasonography performed at 33 weeks gestation confirmed fetal growth restriction, oligohydramnios, and communicating ventriculomegaly with reduced cerebral volume. The mother underwent elective cesarean section at 37 weeks gestation. A female baby was delivered with Apgar scores of 8 at one minute and 10 at five minutes, weight 2050 g, and microcephaly (head circumference 28 cm). The baby had MRI features suggestive of antenatal ischemic encephalopathy and had frequent episodes of focal seizures, which were controlled with clonazepam. Steady but slow growth was reported, with delayed developmental milestones, and the baby is currently undergoing rehabilitation. Since delivery, the mother's thyroid and liver function tests have remained normal.

## Discussion

PTU inhibits the peripheral conversion of T4 to T3. Like methimazole (MMI), it inhibits the utilization of iodine by the thyroid gland. In pregnancy, PTU is preferred to MMI because MMI is associated with a very rare congenital anomaly, aplasia cutis, if administered during the first trimester. However, this risk is negligible and does not persist beyond the critical period of organogenesis [2]. PTU is also preferred to MMI in life-threatening thyrotoxicosis and thyroid storm because of its ability to inhibit peripheral conversion of T4 to T3. It is also reserved for patients who experience adverse effects resulting from MMI or when radioiodine treatment or surgery is not a suitable option [6]. The starting dose of PTU is 100 mg thrice daily [2]. Pregnancy-specific changes affecting thyroid function and iodine metabolism do not influence dosage.

Unlike MMI, the side effects of PTU are not dose-related. About 5% of patients experience cutaneous reactions, arthralgia, and gastrointestinal upset [7]. Agranulocytosis occurs in 0.37% of patients receiving PTU [8]. The frequency of hepatotoxicity ranges from 0.1% to 0.2% [7]. PTU-induced hepatotoxicity can occur at any time during the course of treatment. Its onset is sudden and rapidly progressive [6,9]. Our patient had been on treatment for eight weeks when the diagnosis was made.

The America Thyroid Association and the US Food and Drug Administration (FDA) do not recommend routine monitoring of white blood cell count, liver function, and hepatocellular integrity for PTU-induced liver failure in asymptomatic patients since the onset is usually very rapid. However, these tests are useful in symptomatic patients [6]. Our patient underwent these tests when she complained of jaundice. PTU-related hepatotoxicity is associated with marked elevation in aminotransferase and massive hepatic necrosis on biopsy. These findings were confirmed by histopathologic examination of the liver in our patient.

Therapy consists of immediate cessation of PTU along with expectant management of the potential complications of hepatic failure. A fatality rate of 25% to 50% has been reported in these patients, even though milder cases that resolve uneventfully might not have been reported in the literature [2,10].

In severe cases of end-stage liver failure, liver transplantation may be required [11], though successful treatment with plasmapharesis has also been reported [12]. These treatments are offered only in specialized centers of excellence. As such, our patient could not get treatment locally even if she wished to. The main challenge with liver transplantation in pregnancy is that many medications used in post-transplant immunosuppression may have adverse effects on the fetus, some of which manifested after birth in our case. There are also ethical issues to contend with concerning the likely survival of both the mother and the fetus. A case-control analysis of pregnancy outcomes among liver transplant recipients found most outcomes to be favorable, although there was an increase in rates of miscarriages, pre-term labor, intra-uterine growth restriction, post-partum hemorrhage, and gestational hypertension [13]. To the best of our knowledge, 12 cases of liver transplantation for fulminant hepatic failure between the 13th and 27th weeks of pregnancy have been reported in the literature, with a fetal survival rate of 36% [4,5,13].

## Conclusion

Our case illustrates a very rare complication of a presumed safe drug in a patient requiring a liver transplant and thyroidectomy that echoes the FDA warning on the drug. The unfavorable neonatal outcome may have been due to combined or individual effects of immunosuppressive therapy, prolonged episodes of hypoxia due during assisted ventilation at surgery, or the disease process itself.

## Consent

Written informed consent was obtained from the patient for publication of this case report and any accompanying images. A copy of the written consent is available for review by the Editor-in-Chief of this journal.

## Competing interests

The authors declare that they have no competing interests.

## Authors' contributions

ES managed this patient during pregnancy, provided his professional opinion throughout the management of the patient, obtained consent from the patient for publication of this case report, is the custodian of the patient's records and contributed to the writing of the final manuscript. SW summarized the case, did the literature search, and was a major contributor to the writing of the manuscript. RD also did the case summary by compiling all the medical reports and contributed extensively to the writing of the manuscript and interpretation of the results. All authors read and approved the final manuscript.
